# Study on Climate and Grassland Fire in HulunBuir, Inner Mongolia Autonomous Region, China

**DOI:** 10.3390/s17030616

**Published:** 2017-03-17

**Authors:** Meifang Liu, Jianjun Zhao, Xiaoyi Guo, Zhengxiang Zhang, Gang Tan, Jihong Yang

**Affiliations:** 1 Provincial Laboratory of Resources and Environmental Research for Northeast China, Northeast Normal University, Changchun 130024, China; liumf985@nenu.edu.cn (M.L.); zhaojj662@nenu.edu.cn (J.Z.); guoxy914@nenu.edu.cn (X.G.); 2 Jilin Surveying and Planning Institute of Land Resources, Changchun 130061, China; tangang156@163.com (G.T.); yangjh123@hotmail.com (J.Y.)

**Keywords:** HulunBuir, grassland fire, logistic regression, fire climate

## Abstract

Grassland fire is one of the most important disturbance factors of the natural ecosystem. Climate factors influence the occurrence and development of grassland fire. An analysis of the climate conditions of fire occurrence can form the basis for a study of the temporal and spatial variability of grassland fire. The purpose of this paper is to study the effects of monthly time scale climate factors on the occurrence of grassland fire in HulunBuir, located in the northeast of the Inner Mongolia Autonomous Region in China. Based on the logistic regression method, we used the moderate-resolution imaging spectroradiometer (MODIS) active fire data products named thermal anomalies/fire daily L3 Global 1km (MOD14A1 (Terra) and MYD14A1 (Aqua)) and associated climate data for HulunBuir from 2000 to 2010, and established the model of grassland fire climate index. The results showed that monthly maximum temperature, monthly sunshine hours and monthly average wind speed were all positively correlated with the fire climate index; monthly precipitation, monthly average temperature, monthly average relative humidity, monthly minimum relative humidity and the number of days with monthly precipitation greater than or equal to 5 mm were all negatively correlated with the fire climate index. We used the active fire data from 2011 to 2014 to validate the fire climate index during this time period, and the validation result was good (Pearson’s correlation coefficient was 0.578), which showed that the fire climate index model was suitable for analyzing the occurrence of grassland fire in HulunBuir. Analyses were conducted on the temporal and spatial distribution of the fire climate index from January to December in the years 2011–2014; it could be seen that from March to May and from September to October, the fire climate index was higher, and that the fire climate index of the other months is relatively low. The zones with higher fire climate index are mainly distributed in Xin Barag Youqi, Xin Barag Zuoqi, Zalantun Shi, Oroqen Zizhiqi, and Molidawa Zizhiqi; the zones with medium fire climate index are mainly distributed in Chen Barag Qi, Ewenkizu Zizhiqi, Manzhouli Shi, and Arun Qi; and the zones with lower fire climate index are mainly distributed in Genhe Shi, Ergun city, Yakeshi Shi, and Hailar Shi. The results of this study will contribute to the quantitative assessment and management of early warning and forecasting for mid-to long-term grassland fire risk in HulunBuir.

## 1. Introduction

Grassland fire is one of the most important disturbance factors of natural disturbance to the ecosystem, and poses a very critical problem [1,2,3]. Natural and human factors have a great impact on the occurrence and spread of grassland fires [4], and grassland fire is a major disaster in the world today. It has a great impact and a strong destructive influence on the ecological balance. The area of global grassland affected by fire is 73,619.16 km^2^ [5]. Statistics show that China’s grassland area is approximately 4 × 10^6^ km^2^, accounting for 41.7% of China’s land area, with fire-prone areas accounting for one third and frequent fire area accounting for one sixth [6]. Grassland fire disasters bring huge losses to livestock development and endanger human lives and grassland ecosystems [7].

Grassland fire occurrence is susceptible to weather and climate [8,9,10]. Weather and climate factors exert a great impact on determining the fire regime of a region [11,12,13], affecting the combustibility of fuel through sunshine hours, temperature, precipitation, atmospheric humidity and wind speed directly. Therefore, it could be argued that climate conditions determine the monthly changes in grassland fires. On a long-term scale, the time, intensity and frequency of grassland fire occurrence vary greatly in time and space, and have significant seasonality [14]. The “fire season” refers to the time or season in a region where fire occurred and whose temperature is relatively high, precipitation is relatively low, and combustibles are dry. Local-scale controls on fire regimes are always dominated by regional climate influences [15,16,17].

In the savannah grassland of Africa, most of the fire appears in the dry season (June–August) [18,19]. The occurrence of grassland fire in the north of China mainly appears in the spring (March to June) and autumn (September to November) and the grassland fire season lasts for 7 months of the year [20,21]. The temporal distribution of grassland fires in HulunBuir is closely related to the climate characteristics of this region [22].

Forest fire has been better studied than grassland fire, and research on grassland fire generally has been concentrated on grassland fire hazards [23,24]. Grassland fire potential can be assessed at any scale based on long-term and short-term indices, or with integrated rating systems that include both long-term and short-term variables to produce the potential fire environment [25]. The study on short-term fire weather is more extensive, as is that on climate and fire occurrence, which has examined the spatial and temporal relationships between fire occurrence and climate conditions [26,27,28]. At present, related domestic and foreign research has mostly focused on the fire weather index, which aims to capture the driving effect of climate factors on grassland fire occurrence [29,30,31]. Studies of a fire weather index are usually divided into two parts. Firstly, they integrate the meteorological factors with grassland fire events to establish the fire weather index, which is classified into rating classes in order to interpret the severity of fire events. Researchers around the world have used meteorological indicators to build a large number of fire weather classification models and fire risk indices [13,32,33], though most are focused on forest fire and relatively few pertain to grassland fire. Internationally, the United States and Canada were the first countries to conduct studies on forest fire risk and thus have obtained the most research [34]. The United States developed a national fire risk forecasting system in 1972 and improved it in 1978; it has been widely used throughout the world [35]. Canada began to conduct forest fire studies in the 1920s. The Canadian fire weather indicator system, issued in 1974, is based on a plethora of ignition test and meteorological data, from the theory of moisture balance of combustible materials, through a series of derivations and calculations, and, finally, to fire weather indicators (FWI) [36]. Since the 1950s, forest fire research has been carried out in China, and in the 1970–1980s, more than 10 kinds of fire weather forecasting methods were developed. With the development of GIS technology, remote sensing technology and mathematical modelling techniques, forest fire forecasting has been vastly improved [37]. However, the grassland fire weather index model only reflects the short-term meteorological factors in grassland fire events. Short-term meteorological factors cannot fully explain their effect on grassland fire occurrence, so there should also be medium and long-term scale driving effects of climate factors on grassland fire occurrence. Secondly, researchers employ a larger spatiotemporal scale to study the risk of fire occurrence, fire season, fire intensity and fire behavioral changes caused by climate change [38,39,40]. Climate change plays an important role in temporal and spatial variations in fire seasonality in some regions [2], also affecting the fire frequency, fire season length and fire area [41]. On a large spatial scale, climate exerts a synchronizing and regional control on fire occurrence [42]. In summary, the statistical methods of meteorological factors and fire occurrence events are established by principal component analysis and cluster analysis, and focus on the application of the existing fire weather indexes in grassland fires.

Fire ignition datasets can be obtained from satellite observations [43]. Using the moderate-resolution imaging spectroradiometer (MODIS) active fire products MOD14A1 (Terra) and MYD14A1 (Aqua) and related climate data, the model of fire climate index is established based on the logistic regression method for HulunBuir. On the monthly time scale, the spatial and temporal distribution pattern of fire climate is analyzed in terms of the climate conditions that can affect grassland fire occurrence, which is the basis for the research and analysis of the temporal and spatial variability of grassland fire. This study of the grassland fire climate lays the foundation for the study of the medium and long-term climate with respect to fire and provides a theoretical basis for a medium and long-term early warning and forecasting systems for disaster prevention and reduction of grassland fire occurrence.

## 2. Material and Methods

### 2.1. Study Area

**Figure 1:**
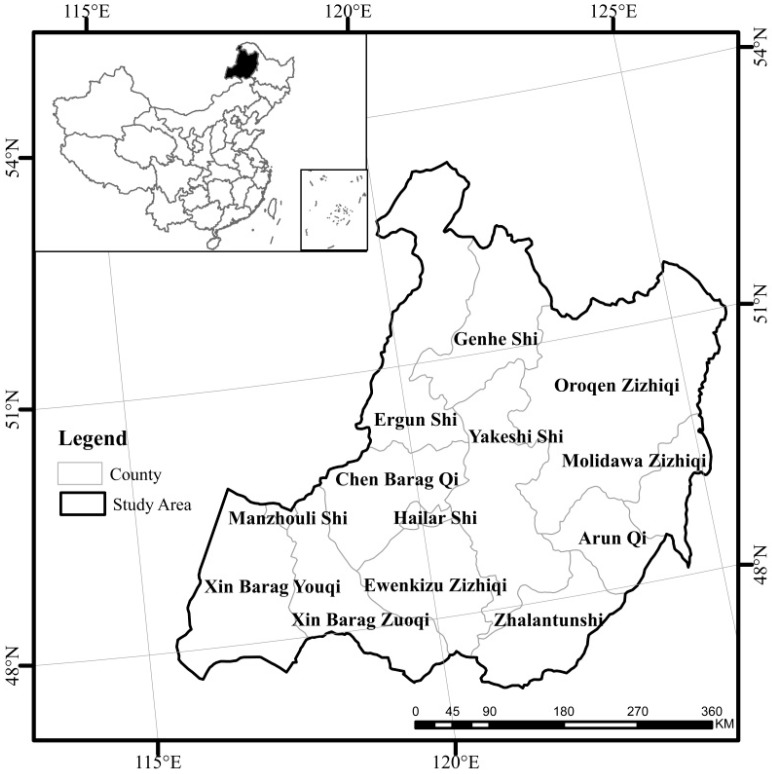
The location of the study area.

The study area is administered by HulunBuir of the Inner Mongolia Autonomous Region in China, which is within 115.22–126.06° E and 47.08–53.23° N. The study area covers an area of 252,948 km^2^, and comprises 13 counties (Figure 1). The topography becomes gradually flatter with a decrease in elevation from the center to the east and to the west. The area of the Grassland in HulunBuir is approximately 83,000 km^2^. The study area has a typical temperate continental monsoon climate; the annual precipitation is approximately 250 mm to 400 mm with a decrease from southeast to northwest, while the average annual temperature is approximately −3 °C to 0 °C with a general decrease in temperature from southeast to northwest. The study area is dry and windy in spring and warm briefly in summer. Temperatures drop quickly and frost begins early in autumn, and winter is long and cold. The aridity indices of April and September are higher than other months. The vegetation in the region is diverse, including meadow steppe, typical steppe and desert steppe from east to west.

### 2.2. Data

#### 2.2.1. Grassland Active Fire Data

The active fire data of MOD14A1 (Terra) and MYD14A1 (Aqua) from 2000 to 2014 are both downloaded from the level-1 and atmosphere archive & distribution system, distributed active archive center and both data products are 8-day composite level 3 fire products [44]. The region included four images, h25v03, h25v04, h26v03 and h26v04, all of which used the MODIS standard sub-frame. The product has a spatial resolution of 1 km and a temporal resolution of 1 day. Each grid cell was capable of daily detection of fire occurrence, and eight-day data were packed into a single folder.

#### 2.2.2. Climate Data

Climate data from nine meteorological stations in HulunBuir for the period from January 2000 to December 2014 were compiled, including the following parameters: monthly average temperature, monthly maximum temperature, monthly average relative humidity, monthly average wind speed, monthly minimum relative humidity, monthly precipitation, monthly sunshine hours, monthly number of days with precipitation greater than or equal to 1 mm, monthly number of days with precipitation greater than or equal to 2.5 mm, monthly number of days with precipitation greater than or equal to 5 mm, monthly maximum wind speed, and monthly maximum daily precipitation.

#### 2.2.3. Land Use Data

To extract the distribution of grassland fire points more accurately, land use data were used to determine the distribution of grassland resources and to remove the non-grassland fire points. Landsat7 TM satellite imagery of August in 2010 was used, with a spatial resolution of 30 m and a time resolution of 16 days. Data were obtained from the Institute of Remote Sensing and Digital Earth, Chinese Academy of Earth Observation Data Sharing Service Web site [45]. Radiation correction, atmospheric correction, projection transformation, mosaic, and cutting were used to process the image data. In the ENVI software, the land use data of the study area were obtained by using the supervised classification method.

### 2.3. Methods

#### 2.3.1. Extraction of Grassland Active Fire Data

The MODIS Reprojection Tool (MRT) is a tool for mosaic, resampling and re-projection of multiple images processed by the NASA website, which can run MODIS data in the JAVA environment. The MRT can be used to select arbitrary bands of MODIS data and to read and process MODIS data in HDF format. By using MRT, the data to be processed can be converted into the projection required by the user, as well as converted to the GeoTIFF or HDF format supported by most software. In this paper, the MRT has been used to mosaic four images every 8 days, and the nearest neighbor method was selected for re-projection.

The MODIS original sinusoidal projection is converted to the Albers area projection, and the original MODIS data in HDF format are converted to the GeoTIFF format. The original data of MOD14A1/MYD14A1 consist of four kinds of fire information: Fire Mask, Quality Assurance (QA), Maximum Fire Radiative Power (FRP), and Scan Sample. The Fire Mask section is used here. The data of MOD14A1 and MYD14A1 with MRT resampling and re-projection are mainly used in the Fire Mask, where each 8-bit unsigned dataset is stored for each daily fire occurrence, and each pixel is assigned a value from 0 to 9 (Table 1).

By using the mask extraction tool in ArcGIS software, the MRT-processed image data are extracted. To produce the image of the study area, HulunBuir’s administrative boundary is used to cut the extracted image.

We extract the data of three levels, 7 (Low Confidence), 8 (Medium Confidence), and 9 (High Confidence), to obtain all the fire points of the 2000–2014 study area.

Thirty-meter grassland land use data from 2010 are used to screen the data to obtain the annual grassland fire points.

#### 2.3.2. Establishment of Fire Climate Model

The grassland fire climate index is used to express the probability of grassland fire occurrence on any day of the month. Therefore, logistic regression was used to establish the fire climate index model. The number and spatial distribution of non-fire points are critical to the assessment of the fire climate index model. In this paper, the spatial distribution of non-fire points is randomly generated in ArcGIS. The number of grassland fire points from January 2000 to December 2010 was collected and counted. There were 132 months from 2000 to 2010, of which 101 months had grassland fire points and 31 months had no grassland fire points. For a month with grassland fire points, an equal number of non-fire points are randomly generated; for a month without grassland fire points, the number of non-fire points is equal to the average number of grassland fire points of 101 months. The probability of the grassland fire points was recorded as 1 and that of the non-fire points as 0.

Spatial distributions of the climate parameters, including monthly average temperature, monthly maximum temperature, monthly average relative humidity, monthly average wind speed, monthly minimum relative humidity, monthly precipitation, monthly sunshine hours, monthly number of days with precipitation greater than or equal to 1 mm, monthly number of days with precipitation greater than or equal to 2.5 mm, monthly number of days with precipitation greater than or equal to 5 mm, monthly maximum wind speed, and monthly maximum daily precipitation from January 2000 to December 2010, were obtained in ArcGIS by using Kriging interpolation. Based on the logistic regression model in SPSS, the stepwise variable approach was adopted to establish the relationship between the random occurrence probability of grassland fire and the climate parameters; the grassland fire climate index model is therefore given as below:

(1) $$P=\frac{1}{1+\exp^{-z}}$$

(2) $$Z=b_{0}+b_{1}x_{1}+b_{2}x_{2}+\ldots +b_{i}x_{i}$$

In this model, *P* is the grassland fire climate index, which represents the probability of the random occurrence of grassland fire in a month; *Z* is a linear combination of independent variables, $x_{i}$ is the value of the $i_{th}$ climate factor; $b_{0}$ is the constant of the model; and $b_{i}$ is the regression coefficient of climate factor $x_{i}$.

#### 2.3.3. Accuracy Assessment of the Model

The receiver operator characteristic (ROC) curve is a statistical method widely used to comprehensively assess the performance of classifiers [46,47]. The area under ROC curve (AUC) for the fire climate index model indicated the model prediction accuracy.

The climate data from January to December of 2011–2014 were interpolated by Kriging to obtain monthly spatial distribution data for each climate parameter, which were then substituted into the fire climate index model, and the spatial distribution of fire climate index in the study area was calculated monthly. The monthly mean value of the fire climate index in 13 counties from January to December of the years 2011–2014 was obtained using statistical tools. We calculated the monthly mean of the fire points in 13 counties from January to December of 2011–2014 and performed a Pearson correlation analysis between the calculated result and the monthly mean of the fire climate index of the corresponding month. The Pearson correlation coefficient can be used to verify the model.

#### 2.3.4. Classification of Fire Climate Zones

Utilizing the ArcGIS software, this paper classified the fire climate index from January to December of 2011–2014 with the equal interval classification method. The fire climate index was divided into five classes, including an extremely low fire climate index zone (*P* < 0.2), a low fire climate index zone (0.2 < *P* < 0.4), a medium fire climate index zone (0.4 < *P* < 0.6), a high fire climate index zone (0.6 < *P* < 0.8) and an extremely high fire climate index zone (0.8 < *P* < 1).

## 3. Results

### 3.1. Distribution of Active Fires

**Figure 2:**
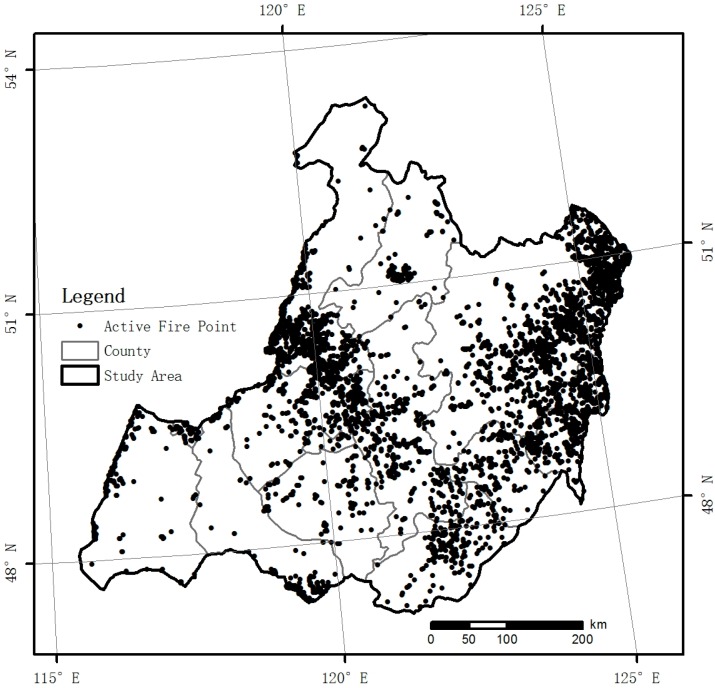
Distribution of grassland active fires in HulunBuir from 2000 to 2014.

After extracting of grassland active fire data, the distribution map of grassland active fire data of HulunBuir from 2000 to 2014 was obtained (Figure 2 and Table 2). For the period from 2000 to 2014, the total number of grassland active fire points was 5565 in the study area. On the inter-annual scale, the number of grassland active fire points in 2000 was the highest, with 913. In 2004, the number of grassland active fire points was the lowest, with 138. On the inter-monthly scale, grassland fires occurred mainly in April, May, September and October; in January and December, the occurrences of grassland fires were the lowest. In the spatial distribution, the occurrence of grassland fires appeared mainly in Oroqen Zizhiqi, Molidawa Zizhiqi, Arun Qi, Zalantun Shi, Chen Barag Qi and Manzhouli Shi; the number of grassland fire occurrences in other counties was relatively low.

### 3.2. Fire Climate Index Model

Of the 132 months in 2000–2010, 101 months had occurrences of grassland fire, and the total number of grassland active fire points was 4274. Because the average number of grassland fire points per month was 42, the number of non-fire points per month was 42, and the total number of non-fire point in 31 months was 1302.

Based on the logistic regression model, the grassland fire climate index model was constructed with monthly average relative humidity (RH), monthly average temperature (TEM), monthly average wind speed (WS), monthly precipitation (PT), monthly sunshine hours (SH), monthly minimum relative humidity (MRH), monthly number of days with precipitation greater than or equal to 5 mm (PD), monthly average maximum temperature (MTEM) as the variables impacting fire climate index, and the regression coefficient of each climate factor in the model satisfies *P* < 0.001. The form of the grassland fire climate index model is given by:

*P* = 1/(1 + exp(−(2.494 − 0.005 × PT + 0.002 × WS − 0.143 × TEM − 0.017 × RH + 0.111 × MTEM + 0.007 × SH − 0.088 × MRH − 0.041 × PD))) (3)

SH, MTEM and WS had positive correlations with the fire climate index. RH, TEM, PT, MRH and PD had negative correlations with the fire climate index. Monthly number of days with precipitation greater than or equal to 1 mm, monthly number of days with precipitation greater than or equal to 2.5 mm, monthly maximum wind speed and monthly maximum daily precipitation had no significant influence on the fire climate index model.

**Figure 3:**
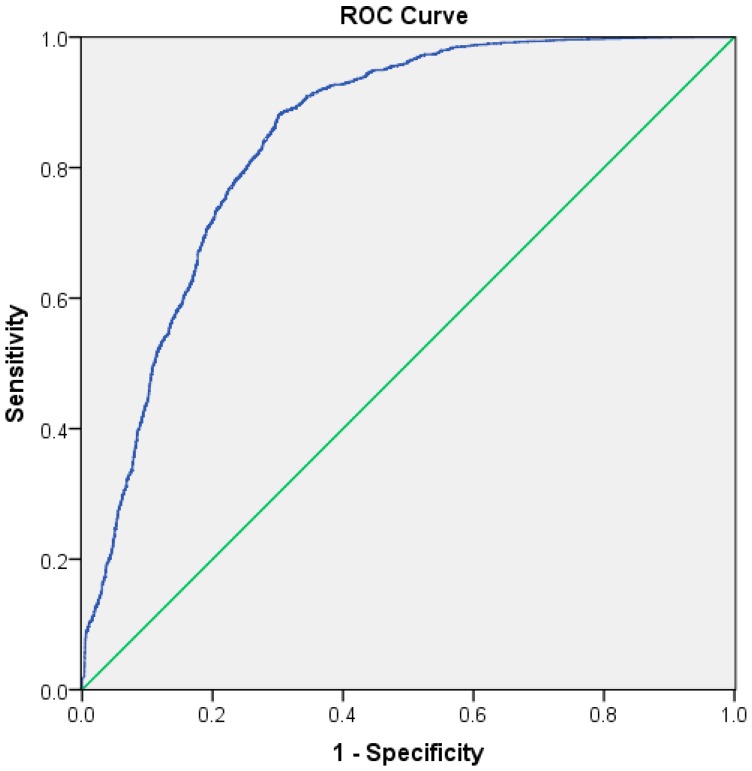
The receiver operator characteristic (ROC) curve of the fire climate index model.

Figure 3 showed the ROC curve of the fire climate index model, and AUC for the fire climate index model was 0.845(95% confidence interval (CI): 0.837~0.950), which indicated an ability of the fire climate index model to predict the occurrence of grassland fire.

To verify the fire climate index model, monthly precipitation, monthly average temperature, monthly average relative humidity, monthly average wind speed, monthly sunshine hours, monthly maximum temperature, monthly minimum relative humidity and monthly number of days with precipitation greater than or equal to 5 mm from January to December of 2011–2014 were subjected to Kriging interpolation and were then substituted into the fire climate index model. The monthly mean values of the climate index of 13 counties from January to December were obtained using statistical tools. We calculated the monthly mean of the fire points in 13 counties in the years 2011–2014, and a Pearson correlation analysis was performed on the monthly mean of the climate index of the corresponding month (Table 3). The Pearson correlation coefficient is 0.578, which indicates that the fire climate index is positively correlated with the number of grassland fire occurrence, and therefore, the fire climate index can be used to express the grassland fire occurrence in HulunBuir.

### 3.3. The Fire Climate Zones of The Grassland

**Figure 4:**
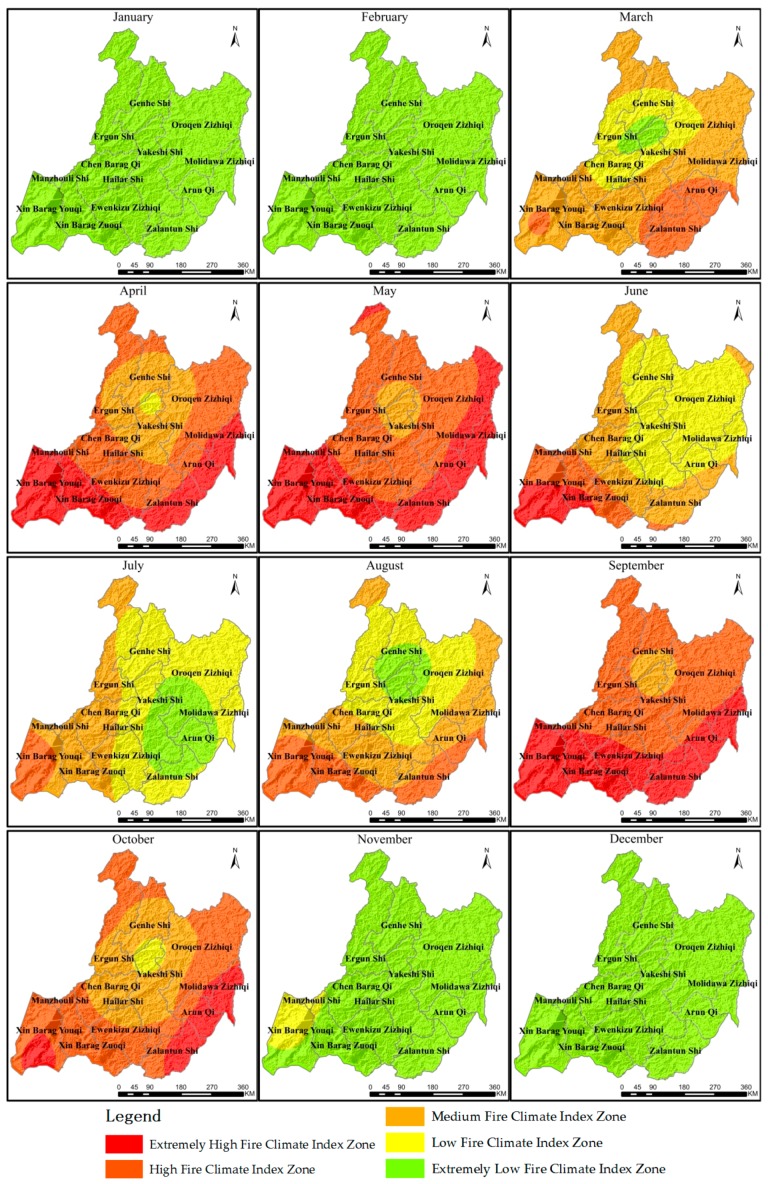
The distribution of monthly grassland fire climate in HulunBuir.

The proportion of the study area in each fire climate index zone in each month of an average year is presented in Table 4 and Figure 4. In 2011–2014, the months with higher fire climate indices were April, May and September, October, and in the other months, the fire climate index was lower. December, January and February were months with an extremely low fire climate index zone. In March, the fire climate index decreased from southeast to northwest, the area proportion of the medium fire climate index zone was 57.10%, and the area proportion of the extremely low fire climate index zone was 4.21%. In April, the fire climate index increased from the center to the surroundings, the area proportion of the high fire climate index zone was 41.98%, and the area proportion of the low fire climate index zone was 1.34%. In May, the fire climate index increased from the center to the surroundings; the area proportion of the high fire climate index zone was 50.44%, and the area proportion of the medium fire climate index zones was 6.71%. In June, the fire climate index increased from northeast to southwest, the area proportion of the low fire climate index zone was 49.33%, and the area proportion of the extremely high fire climate index zone was 7.88%. In July, the fire climate index increased from east to west, the area proportion of the low fire climate index zone was 47.13%, and the area proportion of the high fire climate index zones was 6.69%. In August, the fire climate index increased from the center to the surroundings, the area proportion of the medium fire climate index zone was 35.48%, and the area proportion of the extremely low fire climate index zone was 8.92%. In September, the fire climate index increased from the center to the surroundings, the area proportion of the high fire climate index zone was 53.32%, and the area proportion of the medium fire climate index zone was 5.88%. In October, the fire climate index increased from the center to the surroundings, the area proportion of the high fire climate index zone was 50.89%, and the area proportion of the low fire climate index zone was 3.16%. In November, the area proportion of the extremely low fire climate index zone was 93.20%, and the area proportion of the low fire climate index zone was 6.80%.

On the whole, the zones with higher fire climate index are mainly distributed in Xin Barag Youqi, Xin Barag Zuoqi, Zalantun Shi, Oroqen Zizhiqi, and Molidawa Zizhiqi; the zones with medium fire climate index are mainly distributed in Chen Barag Qi, Ewenkizu Zizhiqi, Manzhouli Shi, and Arun Qi; and the zones with lower fire climate index are mainly distributed in Genhe Shi, Ergun city, Yakeshi Shi, and Hailar Shi.

## 4. Discussion

Monitoring weather plays an important role in the forecast and early warning of fire hazards, but only for the short-term effects of fire weather on the role of grassland fire occurrence, while its effects on the medium- and long-term fire forecast are not obvious. In this paper, on a monthly time scale, we established the fire climate index model, and the relationship between the monthly fire climate index and the occurrence of grassland fire was analyzed; this can be used to improve the fire warning system and provide a scientific basis for mid-to-long-term grassland fire prevention.

From the results of this study, monthly precipitation, monthly average temperature, monthly average relative humidity, monthly minimum relative humidity and monthly number of days with precipitation greater than or equal to 5 mm are negatively correlated with the grassland fire climate index. The greater the precipitation, the greater the humidity, and the higher the moisture content of vegetation, the less likely grassland fire is to occur. In the opposite scenario, grassland fire is more likely to occur. This is consistent with the results of previous studies [27,48].

The correlation between the grassland fire climate index and monthly average temperature is negative, which is not consistent with the previous results [49,50]. HulunBuir has a temperate continental climate, and the precipitation and temperature are positively correlated, i.e., when the temperature increases, the precipitation increases. December, January and February are cold, and the temperature is at its lowest; March, April and May are spring months, and the temperature and precipitation increase slowly; June, July and August are summer months, and the temperature and precipitation are at their highest; and September, October and November are autumn, and the temperature and precipitation decrease. Meanwhile, grassland fire occurred mainly in spring and autumn; in summer, grassland fire occurred less, and in winter, the number of grassland fires was the least. In the study area, the monthly average temperature in April and May was lower than that in June, July and August, but the number of grassland fires that occurred in April and May was more than that in June, July and August. The main reason was with the increase in temperature, the precipitation increased, and the vegetation entered a “green-up” period after the growing season, which increased the water content of the vegetation, so grassland fire was less likely. Therefore, it is reasonable to obtain a negative correlation between the fire climate index and the monthly average temperature in the study area.

The fire climate index of grassland was positively correlated with the monthly maximum temperature, monthly sunshine hours and monthly average wind speed; this result is similar with other findings [8,51]. This phenomenon occurs because long hours of sunshine, high temperatures and high winds can accelerate the evaporation of water, increasing the likelihood of fire.

Within the study area, the zones with higher fire climate index are mainly distributed in Xin Barag Youqi, Xin Barag Zuoqi, Zalantun Shi, Oroqen Zizhiqi, and Molidawa Zizhiqi; the zones with medium fire climate index are mainly distributed in Chen Barag Qi, Ewenkizu Zizhiqi, Manzhouli Shi, and Arun Qi; and the zones with lower fire climate index are mainly distributed in Genhe Shi, Ergun city, Yakeshi Shi, and Hailar Shi. However, there are few occurrences of grassland fire in some zones with higher fire climate index, and the number of fire occurrences corresponding to low fire climate index may be very high, which is also the reason why the Pearson correlation coefficient is not very high. The reason for this is that the occurrence of grassland fire is affected by many factors, but this paper only considered the influence of climate factors on grassland fire and did not consider the factors such as fuel, human activities, vegetation type and land use [19,52,53]. As a result, there are appreciable differences between fire climate index and fire occurrence frequency in some regions.

## 5. Conclusions

HulunBuir, with its temperate continental climate, is the area studied in this paper. On the monthly time scale, based on the logistic regression method, we used the MODIS active fire data product and the relevant climate data to establish a model of grassland fire climate index. Monthly precipitation, monthly average temperature, monthly average relative humidity, monthly minimum relative humidity and monthly number of days with precipitation greater than or equal to 5 mm are all negatively correlated with the grassland fire climate index. The fire climate index of grassland is positively correlated with the monthly maximum temperature, monthly sunshine hours and monthly average wind speed. The results of this study show that the fire occurrence index can be used to represent the fire occurrence in the HulunBuir area during the month, which can provide a scientific understanding of the temporal and spatial regularity of grassland fire for the purpose of forecast and early warning of grassland fire. The fire climate method in this paper provides a way to study the relationship between mid-to-long-term climate change and grassland fire occurrence.

## Figures and Tables

**Table 1:** MOD14A1/MYD14A1 fire mask pixel classes.

Class	Meaning
0	Not processed (missing input data)
2	Not processed (other reason)
3	Water
4	Cloud
5	Non-fire clear land
6	Unknown
7	Low-confidence fire
8	Nominal-confidence fire
9	High-confidence fire

**Table 2:** Monthly occurrences of grassland active fire from 2000 to 2014.

Year/Month	1	2	3	4	5	6	7	8	9	10	11	12
2000	0	0	91	198	55	187	93	29	191	61	8	0
2001	0	1	49	71	6	4	2	3	53	5	12	0
2002	0	16	46	17	9	14	6	43	49	29	70	0
2003	0	15	284	83	289	53	5	0	1	3	0	0
2004	0	0	7	38	5	8	14	34	9	14	8	1
2005	0	0	82	30	11	17	3	34	171	24	0	0
2006	0	0	15	93	110	9	1	6	117	12	1	0
2007	1	22	19	131	12	6	1	42	59	3	0	9
2008	0	16	274	58	15	5	1	7	25	5	23	0
2009	0	0	0	94	29	0	2	1	38	59	6	0
2010	0	0	1	50	28	11	5	9	61	105	16	0
2011	0	0	1	171	6	3	15	40	24	1	1	0
2012	1	2	74	44	27	3	1	15	21	6	9	0
2013	0	0	0	48	75	2	2	1	94	97	29	0
2014	0	1	64	135	12	5	3	10	204	44	2	0

**Table 3:** Pearson correlation of the number of fire occurrences and fire climate index.

Variable	N	Pearson Correlation	Significant Test
Number of fire occurrences & Fire climate index	156	0.578 *	0.000

* Significantly correlated at the 0.01 level (both sides).

**Table 4:** The percent of area of grassland fire climate distribution zones.

	Extremely Low Fire Climate Index Zone (%)	Low Fire Climate Index Zone (%)	Medium Fire Climate Index Zone (%)	High Fire Climate Index Zone (%)	Extremely High Fire Climate Index Zone (%)
January	100	0.00	0.00	0.00	0.00
February	100	0.00	0.00	0.00	0.00
March	4.21	21.40	57.10	17.30	0.00
April	0.00	1.34	24.79	41.98	31.89
May	0.00	0.00	6.71	50.44	42.85
June	0.00	49.33	30.55	12.24	7.88
July	20.31	47.13	25.88	6.69	0.00
August	8.92	33.54	35.48	22.06	0.00
September	0.00	0.00	5.88	53.32	40.80
October	0.00	3.16	31.49	50.89	14.46
November	93.20	6.80	0.00	0.00	0.00
December	100	0.00	0.00	0.00	0.00
